# Evaluation of Lymphovascular Invasion by CD31 Expression in Gastric Adenocarcinoma

**DOI:** 10.30699/ijp.2023.562466.2977

**Published:** 2023-06-20

**Authors:** Seyed Amir Miratashi Yazdi, Elham Nazar

**Affiliations:** 1 *Department of General Surgery, Sina Hospital, Tehran University of Medical Sciences, Tehran, Iran*; 2 *Department of Pathology, Sina Hospital, Tehran University of Medical Sciences, Tehran, Iran*

**Keywords:** Gastric carcinoma, CD31, Lymphovascular invasion, Recurrence

## Abstract

**Background & Objective:**

Lymphovascular tumoral invasion is a typical histopathological feature of gastric carcinomas and supports the recognition of high-risk patients for the recurrence. We aimed to study CD31 expression in diverse subtypes of gastric carcinomas and to show its association with the histopathologic findings of the carcinoma to assess the prognosis.

**Methods:**

This cross-sectional study was conducted on 40 established patients with gastric adenocarcinoma from radical gastrectomy. The patients were classified according to the pathology assessments. Tumoral tissues were assessed by immunohistochemical staining for CD31 expression. Malignant behavior was estimated by histopathological evaluations.

**Results:**

CD31 positivity was described in 23 (57.5%) of all evaluated patients. In assessment of CD31 expression and tumor features presented, no significant association between the CD31 expression and patients’ age, sex, tumor site, size, grade and stage, subtypes of carcinoma, perineural invasion, and also lymphovascular invasion was found. (*P*>0.05).

**Conclusion:**

Lymphovascular invasion may make valuable additional evidence and may be useful to detect gastric carcinoma patients at high risk for recurrence, who could be candidates for more supplementary therapies. However, in our study, CD31 expression did not show any association with the aggressive histopathologic features of this tumor.

## Introduction

Gastric carcinoma (GC) is the main reason of cancer death worldwide, particularly in China, South Korea, and Japan in the East Asian areas. Gastrectomy (total or partial) and regional lymphadenectomy is still the main approach for GC but it has a low free-disease survival rate due to the high rate of recurrence after complete surgery (1). Gastrectomy is only efficient in interfering with the treatment or long-term survival, and lymph node condition is a prominent independent factor for the prognosis prediction. Lymphovascular invasion consists of the infiltration of cancerous cells into the lymphatic or blood vessels, and being close to the tumor is an initial step for the lymph node involvement (2). Angiogenesis in tumors is necessary for development of solid tumors, including GC (3). Tumor extension and lymph node involvement are essential indicators for prognosis of GC. Thus, lymphovascular invasion is an important factor for GC recurrence. Lately, the immune-histochemical (IHC) staining for lymphatic and blood vessels reveal higher sensitivity and specificity than the hematoxylin and eosin (H&E) staining for detection of lymphovascular invasion (4). GC has rich lymphatic flow, and lymph node involvement is frequent. However, an accurate recognition of the lymph node involvement is difficult, and the amount of lymph node involvement is essential for the proper management in the patients with GC after surgery (5). The most accurate prognosis evaluation after surgery, is obtained from the gastrectomy specimen assessment by the International Union Against Cancer/American Joint Committee on Cancer (UICC/AJCC) TNM staging guideline. It was proved that numerous patients classified according to the UICC/AJCC TNM stage, have various survival rates (6). Thus, there is urgent need to introduce new prognostic factors to help recognize the patients with GC, who had high probability of relapse and who may need supplementary therapies after surgery (7). CD31 acts in angiogenesis, detachment of leukocytes, stimulation of lymphocytes, and stimulation of platelets (8). CD31 is a platelet endothelial cell adhesion molecule 1 (PECAM-1), which has a receptor on endothelial cells (9). In this study, we evaluated blood and lymphatic vessels for CD31 expression on endothelial cells. Our study assessed the detection of lymphovascular invasion of CD31 in GC specimens by H&E and IHC staining to show its association with other histopathologic findings.

## Material and Methods

**Study Assessment**

Our cross-sectional study was conducted on 40 patients with established GC from radical gastrectomy who were admitted to the Sina Hospital in Tehran between 2021 and 2022. Excluding criteria were patients whose pathology reports were not according to the College of American Pathologists protocol (10) or did not have appropriate paraffin blocks used for IHC staining.

**Fig. 1:**
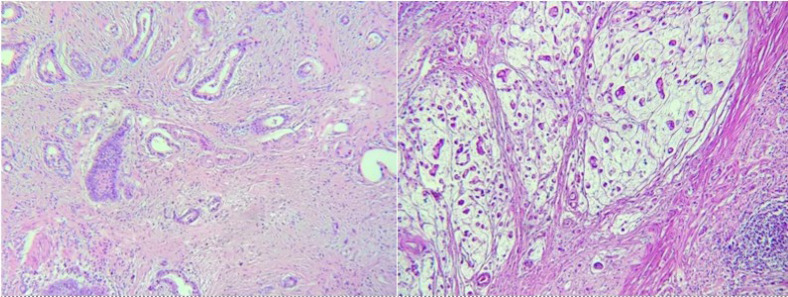
Histopathological examination showed intestinal subtype of the gastric cancer (left) and signet subtype of the gastric cancer (right). (H&E, x100).

**Fig. 2:**
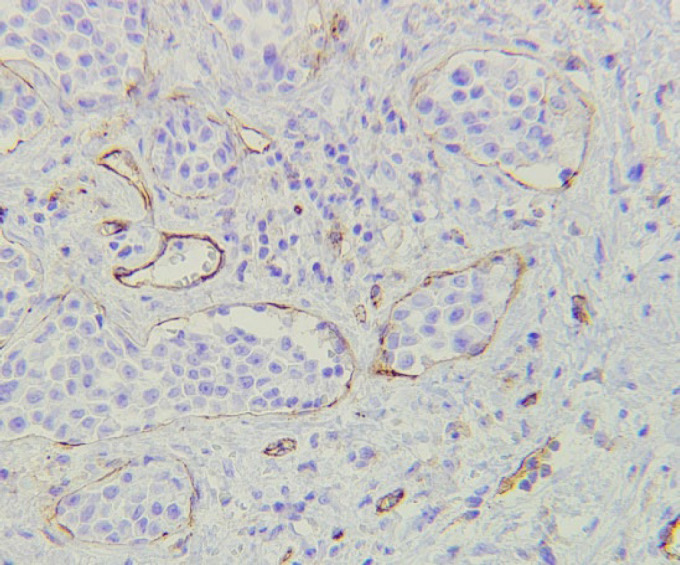
Immunohistochemical staining for CD31 showed lymphovascular invasion by the tumor cells

The tumoral tissues were fixed with 10% formalin and then paraffin blocks were prepared. Tumoral tissue specimens were stained by H&E for assessment of histopathological features, including carcinoma subtypes, tumor grade, tumor extension, tumor size, and presence of perineural and lymphovascular invasions (Figure 1). After staining with H&E, the slides were inspected and a proper zone on each slide was chosen and examined by IHC staining for CD31 marker expression (Figure 2). The association between CD31 expression and malignant histopathological behaviors was assessed, and the sensitivity of lymphovascular invasion by H&E and IHC staining by CD31 was compared in the GC specimens. 

**Statistical Analysis**

For statistical analysis, quantitative variables were accessible as mean ± standard deviation (SD), and categorical variables were revealed by rate (ratio). Continuous variables were linked by the *Mann-Whitney* and *t-tests* when the statistics did not seem to have regular spreading. The categorical variables were linked by the *Fisher’s exact* and *Chi-Square* tests if obligatory. P-values≤0.05 were considered statistically significant. Also, the statistical investigation was done by the statistical software SPSS version 23.0 for Windows (IBM, Armonk, New York). 

## Results

In our study, 40 patients with an established GC diagnosis were included. The median age of the GC patients was 63.20±10.71 years (39 to 84 years), with a high percentage in men (75%). The demographic data are shown in Table 1. The carcinoma subtype and signet subtype of GC were 82.5% and 17.5%, respectively. Forty percent of GCs were considered as grade III. The most GC site of involvement was antrum (50.0%) followed by cardia (27.5%). Perineural invasion was found in 42.5% of the GCs and lymphovascular invasion was found in 45.0%. The average tumor size was 4.31±2.61 cm.

**Table 1:** The baseline characteristics

Gender	
Male	30 (75.0)
Female	10 (25.0)
Mean age, year	63.20±10.72
Subtype	
Intestinal	33 (82.5)
Signet	7 (17.5)
Grade	
I	13 (32.5)
II	11 (27.5)
III	16 (40.0)
Location	
Antrum	20 (50.0)
Body	7 (17.5)
Cardia	11 (27.5)
Fundus	2 (5.0)
Perineural invasion	
Positive	17 (42.5)
Negative	23 (57.5)
Lymphovascular invasion	
Positive	18 (45.0)
Negative	22 (55.0)
Stage	
I	5 (12.5)
II	6 (15.0)
III	23 (57.5)
IV	6 (15.0)
Mean size, cm	4.42±2.98
CD31 expression	
Positive	23 (57.5)
Negative	17 (42.5)

Overall, CD31 expression was evident in 23 (57.5%) of all the tumoral tissue specimens. CD31 expression in males was higher than those in females, with a mean age of 63.20±10.72 years. Also, CD31 expression was noted to be higher in those with intestinal type, or those located in antrum, or tumors with grade III. However, evaluation of the association between CD31 positivity and tumor features exhibited no noteworthy correlation among the CD31 positivity and patients’ age, sex, tumor site and size, subtypes of carcinoma, and also presence of lymphovascular invasion using H&E staining (*P*>0.05) (Table 2).

**Table 2:** CD31 expression according to the baseline characteristics

Item	Expression rate	P value
Gender		0.853
Male	17 (56.7)	
Female	6 (60.0)	
Mean age, year		0.289
≤60 years	12 (66.7)	
>60 years	11 (50.0)	
Subtype		0.983
Intestinal	19 (57.6)	
Signet	4 (57.1)	
Grade		0.918
I	9 (69.2)	
II	3 (27.3)	
III	11 (68.8)	
Location		0.830
Antrum	12 (60.0)	
Body	3 (42.9)	
Cardia	7 (63.6)	
Fondus	1 (50.0)	
Perineural invasion		0.073
Positive	7 (41.2)	
Negative	16 (69.6)	
Lymphovascular invasion		0.822
Positive	10 (55.6)	
Negative	13 (59.1)	
Stage		0.377
I	3 (60.0)	
II	5 (83.3)	
III	13 (56.5)	
IV	3 (33.3)	
Mean size, cm		0.185
≤5 cm	18 (64.3)	
>5 cm	5 (41.7)	

Test: Chi-Square test or Fisher’s exact test.

## Discussion

GC is still a challenging disease and is the most common reason for the cancer-associated death (11). GC progression is a multistep process with genetic and epigenetic changes (12). The mechanism of GC development and metastasis has great significance due to varied disease-free survival frequencies in the patients with similar histopathologic findings (13). There are some helpful prognostic markers for GC (14). In the GC tissues, the lymphatic and blood vessels density was shown to be higher compared to the normal tissues, immunohistochemically (15). The character of lymphovascular invasion in the development and metastasis of GC has been considered over the last few years. Our study assessed the lymphovascular invasion by the tumor cells with H&E staining compared to the CD31 expression using IHC staining as a blood vessel marker and its association with the other histopathologic findings to evaluate the outcome of the patients with GC and predict the possibility of lymph node metastasis. Lymphovascular invasion referred to as blood or lymphatic invasion involves cancerous cells in the lumen of the vessels, which leads to the circulating tumor cells. Lymphovascular invasion is a frequent histopathological feature in many human cancers and has been revealed to be related to high frequency of relapse and poor outcomes in the patients with breast, lung, colon, and renal malignancy (1). A retrospective study advised that exact examinations for lymphovascular invasion in GC revealed valuable data for recognition the patients at high risk for recurrence and those who may be appropriate candidates for supplementary therapy (16). Lymphovascular invasion is recognized as an independent factor for the prediction of lymph node involvement in GC. However, the finding of lymphovascular invasion using H&E staining is usually difficult. IHC staining for blood vessel markers is advised to identify any lymphovascular invasion in GC (17). Therefore, our study was conducted focusing on this issue. 

Mohamed *et al.*, study showed colorectal cancer patients with high expression level of CD31 had poor outcomes and established that IHC markers might be a suitable choice for categorizing patients into low- and high-risk groups (18). Sun *et al.*, showed that CD31 expression was related to metastasis, local invasion, tumor staging, and colorectal cancer (19). Huang *et al.*, evaluated CD31 and CD105 expression in the GC samples and found CD105 as better marker compared to CD31 for prediction of prognosis. CD105 expression is connected to the aggressive behavior of GC. Thus, they advised IHC staining for identification of lymphovascular invasion to precisely evaluate the tumor stage and prognosis. Tumoral cells invade blood vessels close to the tumor in advanced cancers. Thus, lymphovascular invasion is the essential phase of tumor spreading and metastasis in numerous types of cancer. The predictive value of lymphovascular invasion in GC was previously considered in a few studies without attaining an agreement (20). In our study, we did not reach any significant finding or any association with histopathologic features due to aggressive tumors. According to the recent Japanese strategies in GC, lymphovascular invasion is not clinically valuable evidence for the prediction of outcome (21), which is parallel to our discoveries. Dicken et al., described the lymphovascular invasion as a useful marker for the prognosis and related it to the progressive behavior, which was the motivation for some clinicians to recommend that lymphovascular invasion must comprise in risk categorization (22). Scartozzi *et al.*, advised that lymphovascular and perineural invasion were significant features prompting poor outcomes in the GC patients and affecting disease-free survival, which were also dependent on previously recognized prognostic factors, including tumor extension (23). Pak *et al.*, studied 66 patients with GC and showed that peritumoral was more important than intra-tumoral lymphovascular invasion and had a significant role in lymph node involvement (24). However, recent studies concerning the association between lymphovascular invasion and disease-free survival in the patients with GC are debated. Some studies informed that lymphovascular invasion was meaningfully accompanied by the relapse and poor prognosis with lymph node involvement. However, other reports described lymphovascular invasion did not influence the outcome or lymph node involvement, especially in early GC. Thus, with these various consequences on this subject, more studies are required to improve our acknowledge of the prognostic value of lymphovascular invasion and help for supplementary treatment (25). Araki *et al.*, reported that lymphovascular invasion was a significant factor of the recurrence and survival rate in the GC patients with no positive lymph node, and these patients benefit from supplementary therapy after gastrectomy when the lymphovascular invasion is present (26). Therefore, more prospective studies are required to approve our results. Lymphovascular invasion in GC tends to have poor outcomes, particularly in advanced stages with lymph node involvement (21). Lymphovascular invasion should be evaluated in the gastrectomy specimens carefully (27). Angiogenesis suppression in cancerous cells has a potential role in GC treatment (28). Also, galectin-1 is related to CD31 expression and promotes angiogenesis in GC and may be a target for the anti-angiogenic therapy (29). Anti-angiogenic therapies for GC are respected for the long-term administration with no drug resistance (30). In our study, evaluation of CD31 expression in different subtypes of GC suggests that the IHC expression of CD31 is not associated with the histological aggressive findings, and evaluation of lymphovascular invasion through H&E and IHC (using CD31 expression) shows no significant difference. These data may be related to the quite small sample size and other possible factors, including patient characteristics or antibody specifications. In addition, supplementary studies to recognize new therapeutic agents targeting CD31 would be recommended.

## Conclusion

Many studies have revealed that lymphovascular invasion has the main role in tumor development and metastasis. IHC CD1 staining for assessment of lymphovascular invasion might be helpful if the lymphovascular invasion is questionable, and may offers valuable data for GC patient management. Further studies with large samples and new markers for identification of lymphatic or blood vessels would be recommended.

## Ethics

This research study was conducted retrospectively from data obtained for the clinical purposes by the 1964 Helsinki Declaration and its amendments.

Written informed consent was obtained from the patients for the publication of this case report and accompanying images. A copy of the written consent is available for review by the Editor-in-Chief of this journal on request.

## Data Availability Statement

Data will be made available on request.

## Conflict of Interest

None.
